# Miscellaneous Contributions to Pathology and Therapeutics, Being a Series of Original and Practical Papers on Rickets, Hydrocephalus, Impotence, and Sterility, Pulmonary Apoplexy, and Hemoptysis

**Published:** 1845-01

**Authors:** 


### Art. XXI.

*Miscellaneous Contributions to Pathology and Therapeutics, being a series of Original and Practical Papers on Rickets, Hydrocephalus, Impotence, and Sterility, Pulmonary Apoplexy, and Hemoptysis.* By J. R. Smyth, M.D.—*London*, 1844. 8vo, pp. 342.

This volume is a collection of papers on the subjects set forth in the title-page, many of which have appeared before in weekly medical journals. Cases are given and commented upon. The first contribution is in rickets which contains no practical matter of any worth. The practice recommended is judicious, but has been laid down more clearly and concisely by most systematic authors. The author speculates on a connexion between rickets and disease of the liver, but gives no dissections to prove it. The next paper is chronic hydrocephalus. One case is detailed in which the writer resorted to tapping with temporary amendment, but the child ultimately died. After Dr. Watson's masterly analysis of this operation in his 'Lectures on the Practice of Physic,' these crude and half-digested remarks seem especially useless. The existence of a cerebral murmur in hydrocephalic affections of children. This is asserted was first noticed by Dr. Fisher of Boston, whose observations were given in the Thirteenth Number of this 'Review,' in January 1839, page 268. The existence of this sound Dr. Smyth became aware of before he had read Dr. Fisher's paper. He gives an analysis of an essay by Dr. Whitney, published in the 'American Journal of the Medical Sciences,' for October last, in which it is asserted that a murmur or bellows' sound is audible in the head, in congestion, inflammation, compression, and organic diseases in the brain, in hydrocephalus, ossification of the arteries, and excessive general anæmia. A case by Dr. Whitney is given in which aneurism of the basilar artery gave a purring vibratory thrill, heard over the whole cranium.

The next contributions are on impotence and sterility, in which the important relation between the mental well-being of the individual, and a healthy state of the organs of reproduction, and the connexion between abuses of their function, and the failure both of bodily and of mental health, are strongly insisted on. It seems to be the impression of Dr. Smyth and of similar writers that the want of written essays on this subject by men of reputation, is a proof that the facts are not duly recognized. But is this the case? Can any man of ordinary observation be unaware of this fruitful source of mischief? Is not the silence rather a proof that even in this country those whose minds are cultivated and refined by education rather shrink from committing to paper their whole views on a subject which they cannot unveil without doing violence to their best feelings and to their own self-respect? They are content to indicate and allude to such causes in a way which no one who reflects duly, can pass over or misunderstand, and they seem with one accord to leave to pamphleteering quacks the dirty work of revealing in print the private confessions of the most humiliating weaknesses and vices of human nature. For your quack has no such difficulties. It is not that he sees further; but no decency, shame, or compunction, hinders him from exposing all of his most secret surmises and suspicions, or the most secret revelations of his patients; and, led by the joint influence of his own pruriency and love of gain, he concocts his sealed volume, or cunning advertisement, to catch and to delude those whose weaknesses he is so well aware of. The difference between the two lies in the mode in which the subject is treated. The man of true science and taste treats an uncleanly and impure subject with brevity and purity. The quack never recollects that "want of decency is want of sense." The difference is as great as exists between one man who should throw the light of a lamp upon a dung-heap to indicate its presence, and another who, not content with this, should also stir it up and enjoy its reeking vapours.

Without putting the present in this last class, we regret that he approaches it too nearly either for the dignity of science, or for his own reputation. For, like the pamphleteers, he reprints long and disgusting letters full of minute confessions, with hypochondriacal twaddle, and weak flattery of the doctor, which such patients indulge in; and there is throughout a fulness and prolixity of uncleanliness, which men of good taste, to say no more, eschew.

The letters of one patient, a medical person, we regret to say, exhibit a callous viciousness indicating a most degraded moral condition, and they are printed without a word of comment! Nor is the mode of treatment sufficiently new to excuse either the mode in which the subject is treated, or the reprint of these papers which have been previously circulated in a weekly journal. The treatment of the cases given is judicious. They are treated with ordinary remedies according to the individual symptoms of each—in fact just as all judicious practitioners do treat diseases—one with alteratives, others with iron and tonics, &c.

The style of the whole volume is loose, verbose, and discursive, "that easy writing which is such hard reading."

